# Novel method for scalable synthesis of wollastonite nanoparticle as nano-filler in composites for promotion of anti-corrosive property

**DOI:** 10.1038/s41598-021-81875-4

**Published:** 2021-01-28

**Authors:** Rouholah Dordane, Mohammad Mahdi Doroodmand

**Affiliations:** Department of Chemistry, Shiraz University, Shiraz, Iran

**Keywords:** Analytical chemistry, Chemical engineering

## Abstract

This study is focused on novel anti-corrosive support. This coating is based on the mixed matrix (*MM*) including epoxy and its hardener as thermoset polymer, melamine–formaldehyde as the organic phase, activated graphite as both inorganic and conductive phases, as well as wollastonite nanoparticles as filler. The wollastonite nanoparticles are synthesized by the new and novel method as suitable etching using hydrofluoric acid, “*HF*” at room temperature (25 ℃). The synthesized wollastonite nanoparticles are then adapted as a suitable filler during the formation of a new type of MM anti-corrosive coating for the preservation of metals form any corrosion. According to the results, during coating the MM, significant positive characteristics such as enough positive open circuit potential (*OCP*), small enough (*i*_*corr*_), low cost, significant chemical/mechanical stability and acceptable flexibility are observed. Based on to the weight-loss analysis test on the MM-modified stainless steel during a 42-day time interval, the corrosion rate % is decreased from 74.86 to 0.34. In addition, the electrochemical impedance spectroscopy reveals major enhancements in the double-layer resistance and solution resistance of the cell system. Based on the electrochemical measurements, noticeable reduction and enhancement are observed in the correction rate and potential, respectively, during introducing hard corrosive conditions such as NaCl (3.0%, w/v) and HCl (1.0 mol L^−1^) environments that reveal the acceptable anti-corrosive behavior of the synthesized MM. The introduced MM is therefore considered as low cost, safe, eco-friendly, industrial-justified anti-corrosion support.

## Introduction

Wollastonite is one of the natural mineral substances in white and is a type of calcium silicate (CaSiO_3_). In general, wollastonite is known as an industrial material and is used to make materials such as cement, glass, brick, and tile in the construction industry. Wollastonite consists of four categories: monocalcium silicate (CaSiO_3_), dicalcium silicate (Ca_2_SiO_4_), tricalcium silicate (Ca_3_SiO_5_), and tricalcium disilicon heptaoxide (Ca_3_Si_2_O_7_)^1^. This compound is produced in both forms of fiber and powder, plastics and paints, besides its suitability to be selected as a good alternative to the asbestos^1^.

Wollastonite in addition to its mentioned applications, can also have other useful applications. After “*World War II*”, one of the main requirements of some different countries was the treatment of patients during requiring artificial limbs. These bone-binding materials are therefore named as “*Bioactive Materials*”^2^. Over the past decades, scientists and doctors have tried to synthesize a micro composite, at which an assembly of apatite small particles is effectively reinforced by β-wollastonite (CaO∙SiO_2_) with a silicate chain structure during the crystallization of an MgO–CaO–SiO_2_–P_2_O_5_ glass. The results of this work confirmed that, the synthetic micro-composite, in addition to its bioactive behavior, has processed high mechanical strength and is, consequently, good consistent with body conditions^3^.

During the last decade, in the laboratory scale, it has polycrystallized ceramic materials based on pseudowollastonite (β-CaSiO_3_), which is a hydroxyapatite (HA) surface layer during exposing to simulated body fluid (SBF) during making biomaterials. These materials are used as potential implants in rats and dogs^2^. Given the widespread applications of wollastonite as a precursor for production in various fields such as cement, biomaterials, additive of mechanical strength, ceramics, and so on, the introduction of a new synthetic method of wollastonite nanoparticles with well-defined morphology is important. For this purpose, various techniques are used such as precipitation, sol–gel, solid-state reaction, etc.^2^.

Chemical methods have previously been used, such as hydrothermal processes for the production of wollastonite-based composites. For example, fibers [Ca_5_ (Si_6_O_16_) (OH)_2_·4H_2_O] with 1.0 µm average diameter are prepared under the hydrothermal process^4^. The microemulsion not only can serve as a nano-reactor to control the particle diameter and size distribution in the processing reactions, but also, inhibits the excess agglomeration of particles. However, because of problems like high cost, expensive methodology, and hard conditions for implementing, the microemulsion method is not widely applicable at the industrial scale^5^.

Another method for the synthesis of wollastonite is the crystallized wollastonite with no fusion from a natural material with high enough calcium carbonate and silica content. The present invention is related to a process for crystallized wollastonite synthesis from a natural material using a high content of calcium carbonate and silica^6^.

Wollastonite with cylinder structures has also been prepared using green materials such as rice husk ash as the source for SiO_2_ and limestone, as well as the precursor of CaO by the sol–gel method. For this purpose, briefly, rice husk ash and CaO powders are mixed in the sol–gel matrix using the calcining process^7^. In addition, wollastonite powders have been synthesized by microwave-assisted solid-state reaction. It can be used from a bio-solid waste egg-shell using SiO_2_ as the starting materials during starting wollastonite formation at 800 ℃, resulted in obtaining a single-phase after 10 min heating at 1100 ℃. At this condition, the use of microwave heating lowers the processing temperature and time of the wollastonite synthesis^4^.

Typical industrial methods for the synthesis of wollastonite include the hydrothermal, the solid-state, etc. These processes are carried out in a closed compartment under high vapor pressure, elevated temperature, expensive materials and so on. They also involve the use of special equipment(s) to produce high tonnage with no desirable value. These reasons also demand the scientist to present a new method for producing wollastonite^8^. To solve these problems, hereby in this study, we have used a novel etching process with hydrofluoric acid for wollastonite production.

For this purpose, hydrofluoric acid is the only etchant, which attacks amorphous SiO_2_, quartz, or glasses at a significant high etch rate. Therefore, due to the presence of different problems such as temperature, pressure, toxic raw material, expended materials, etc., the introduction of a low cost new method with possibility to largly synthesize the wollastonite is demanded. Comapatibity of these compounds can play role as flexibale coating with siginificant properties, especially corrosion protection. The corrosion protection by immobilizing these coatings can be used, due to several advantages over other methods such as flexibility, simplicity, cheapness, no need to any complex equipment(s), and refinement.

These coatings include a wide range of materials such as colored reagents, metal supports^9^, organic substrates, and polymeric compounds. However, among these reagents, mixed matrix (*MM*) materials are considered as one of the most applicable polymeric coatings, since they include homogeneous mixture of organic, inorganic, conductive phase, and filler phases. These compounds are so porous that are often utilized in different parts of science, especially in the adsorption and desorption of gas mixtures during gas separation process^10,11^ and anti-microbial medium in medicine^12^. These properties are attributed to the presence of both organic and inorganic phases in a matrix. In addition, well-defined cracks (gap), formed during introducing the filler phase, not only promotes the MM flexibility, but also controls the mass transfer rate of different reagents^13^.

It seems that, these characteristics also would control the charge and mass transfer mechanisms in the electrochemical systems. In such a way, the MM can delay the charge and mass transfer phenomena at active corrosion sites to slow down the electrochemical reactions^7^. Specifically, it would make high enough resistance against corrosion process. Clearly, suggestion of adequate fillers such as wollastonite, due to its special characteristics would majorly control the challenges related to the corrosion process. At this condition, factors such as synthetic scale, safety, cost, purity, well-defined structure, and size of the wollastonite granules are important. In this report for the first time, a novel method is introduced to synthesize the wollastonite nanoparticles along with its use in the fabrication of a novel MM of Epoxy/Melamine Formaldehyde/Graphite composite to protect the metallic supports against any corrosion^14^.

## Experimental

Standard materials for the synthesis of wollastonite nanoparticles have been used based on the etching effect of HF. Wollastonite characterization was performed by methods such as X-ray diffraction (XRD, Bruker, Type D8-ADVANCE), thermo-gravimetric analysis/differential scanning calorimetry (*TGA/DSC*, Metrohm), high resolution-transmission electron microscopy (HR-*TEM,* Zeiss EM10, US), and Fourier-transform infrared spectrometry (*FT-IR,* Shimadzu 8300 Series, Japan). In addition, the corrosion behaviors of some supports such as iron, steel (Type: 307, Black Stud, China, and ceramic (China Ceramics Co., Ltd.) during the modification with anti-corrosive materials were characterized by methods like linear polarization (*Toffel plot*), electrochemical impedance spectroscopy (*EIS*, µ3AUT70980), cyclic voltammetry (*CV*, µ3AUT70980), weight-loss measurements, energy-dispersive XRD (*EDS*, TESCAN-Vega 3), scanning electron microscopy (*SEM,* TESCAN-Vega 3, 25 kV) and optical microscopic (TESCAN-Vega 3, 100× mag.) imagings.

### Materials and methods

Analytical grades of NaF, CaF_2_, HCl, HNO_3_, HBr, HI, HIO_4_, H_2_SO_4_, H_3_PO_4_, CH_3_COOH and silica gel (SiO_2_, purity: 100%, w/w), (Merck Company) were used to synthesize the wollastonite nanoparticle using sonication process at room temperature. Solutions were prepared using deionized water. The iron and stainless steel samples were used for the corrosion study. These chemical compositions were tested by Quantometry (Foundry Master Smart, US), which were reported in Supplementary Table 1 (see Supplementary Table 1, Supplementary Information). Epoxy resin, hardener, acidic melamine–formaldehyde (Fars resin manufacturing company, Shiraz, Iran), and graphite (5 × 5 cm, Sinchem, Tehran, Iran) were considered as the component of the MM coating. It is also emphasized o the lack of any unexpected, new, and/or significant hazards or risks associated with this reported work.

### Characterization of the synthesized wollastonite nanoparticles

Factors having important effects on the size and purity of the synthesized wollastonite nanoparticles included: type of acid, sonication, the concentration of acid, the ratio of CaF_2_:HCl:SiO_2_, and the time duration of the introduction of acid to the solid mixture. In this study, the purity, as well as the efficiency of the production of the synthesized wollastonite, was selected as the main important factor during the synthesis of wollastonite. The optimizations were achieved by a one-at-a-time method.

### Formation of mixed matrix anti-corrosive support

To form the mixed matrix, it was initially needed to weight each component of the mixture including epoxy resin, synthetic wollastonite, graphite, and acid melamine–formaldehyde precisely with weight percentages of 65.0, 15.0, 10.0, and 10.0% (w/w) to generate a soil composite. After that, the synthesized composite was handy ground to form a uniform and homogeny solid powder. Finally, this composite was modified and solidified with 30.0% (w/w) hardener (ammonia hardener) to generate a MM paste with small viscosity. The synthesized MM was then directly coated and introduced on the surface of a metal rod (as an electrode) with thickness as small as 0.2 mm. The modified electrode was then baked for 3.0 h inside the vacuum oven at 200 ℃.

### Electrochemical measurements

Electrochemical tests were carried out with a µ-AUTO LAB-µ3AUT70980. The cell arrangement was a conventional three-electrode system including platinum (Diameter: 1.0 mm) as a counter electrode, Ag/AgCl (Sat’d Cl^−^) as the reference electrode, and test material (*F*e) macro-electrode rod (Diameter: 3.0 mm, Shiraz, Iran) as working electrode with a circular diameter, whose sides were insulated using an insulating thermal varnish (Diameter: 3.0 mm Suzhou Volsun Electronics Tech. Co., Ltd., China). Linear polarization curves were recorded using a potentioastat at the scan rate of 1.0 mV s^−1^, in the range of + 250 to − 250 mV versus the open circuit potential (*OCP*). Impedance spectroscopy was measured over a frequency range of 0.1 MHz to 0.1 Hz with an amplitude of + 10.0 mV peak-to-peak using an AC (alternating current) signal, vs. Ag/AgCl electrode. The surface of the iron rod was analyzed after the corrosion tests using the SEM imaging.

### Weight-loss measurements

Weight-loss-measurements were performed on a steel plate (1.0 × 3.0 cm) coupons in 1.0 mol L^−1^ hydrochloric acid solution (100.0 mL) at room temperature before and after coating with the synthesized MM using a gravimeter (Mettler, AT261). Weight-loss of stainless steel plate coupons was noted after an immersing period of 42 days, sequentially according to the Eq. (1) reported below:

1 $$ C. R \left( \% \right) = \frac{{{\text{W}}1 - {\text{W}}2}}{{{\text{W}}1}} \times 100, $$

where *W*_1_ and *W*_2_ are the weight of mild steel before and after immersion in the corrosive environment and *C. R* is the percentage of corrosion rate^8^.

## Results and discussion

**Figure 1:**
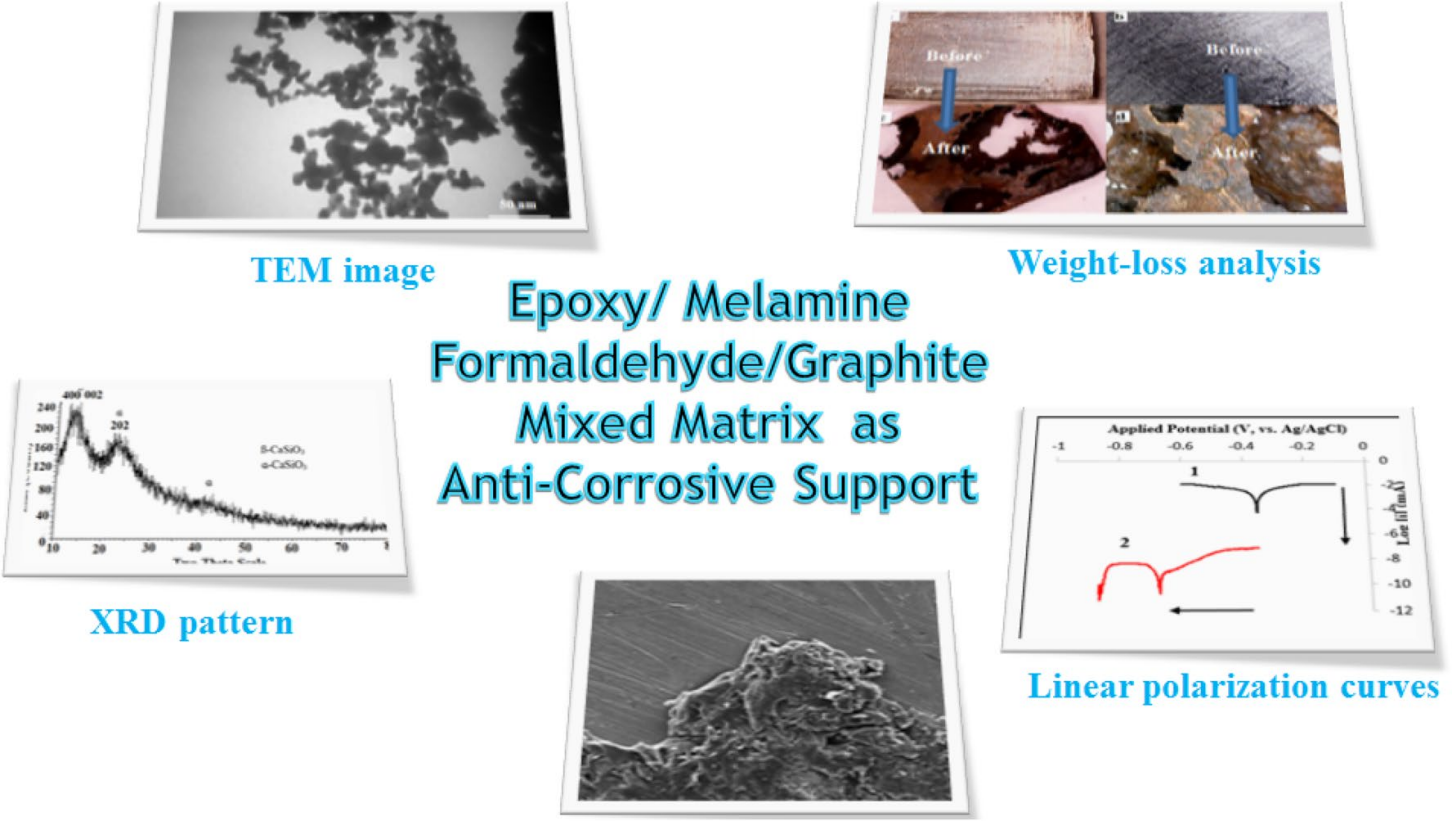
Schematic of the introduced wollastonite synthetic method for the corrosion process.

Schematic of the introduced wollastonite synthetic method for the corrosion process has been graphically shown in Fig. 1. Detail of each step has been described and discussed in detail in the following sections.

### Synthesis of wollastonite nanoparticles

In this experiment, a new synthetic method was introduced for large-scale generation of wollastonite nanoparticles. This process was based on the sonochemical process during the in-situ generation of HF as the SiO_2_-etchant, via mixing of CaF_2_ and SiO_2_ as the initial precursors according to the following three-step process:

- *Step #1* Mixing CaF_2_:HCl:SiO_2_ with molar ratio of 3.0:12.0:1.0.
- *Step #2* Dropwise addition of HCl (0.1 mol L^−1^) into the solid mixture.

According to the following reactions (Eqs. 2 and 3), we have H_2_[CaCl_4_] and H_2_[SiF_6_] as the intermediate medium:

2 $$ {\text{CaF}}_{{2}} + {\text{ 4HCl}} \rightleftharpoons {\text{H}}_{{2}} \left[ {{\text{CaCl}}_{{4}} } \right] \, + {\text{ 2HF,}} $$

3 $$ {\text{SiO}}_{{2}} + {\text{ 6HF}} \rightleftharpoons {\text{H}}_{{2}} \left[ {{\text{SiF}}_{{6}} } \right] \, + {\text{ 2H}}_{{2}} {\text{O}}{.} $$

*Step #3* H_2_[SiF_6_] is then reacted with H_2_[CaCl_4_], during the formation of CaSiO_3_ as the wollastonite nanoparticles. The overall reaction of the synthesized of wollastonite nanoparticle is as follows (Eq. 4):

4 $$ {\text{H}}_{{2}} \left[ {{\text{SiF}}_{{6}} } \right] \, + {\text{ H}}_{{2}} \left[ {{\text{CaCl}}_{{4}} } \right] + {\text{ 3H}}_{{2}} {\text{O}} \rightleftharpoons {\text{CaSiO}}_{{3}} + {\text{ 6HF }} + {\text{ 4HCl}}{.} $$

At the end of this stage, the resulting medium is centrifuged (Thermo Fisher Scientific—US, 5000 rpm) for 15 min. to separate the nanoparticles from the suspension. Meanwhile, washing the solid phase was needed for 2 or 3 times using the deionized water (10.0 mL) to remove any probable impurities from the surface of the synthesized nanostructure.

### Effect of acid

**Figure 2:**
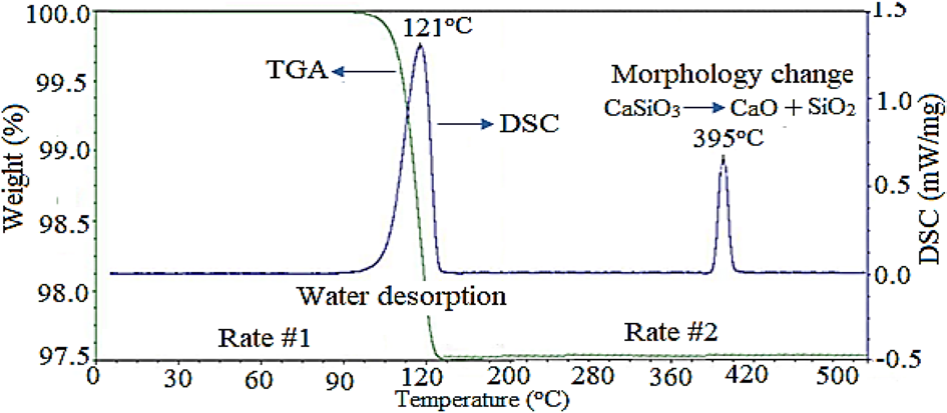
TGA and DSC curves of the synthesized wollastonite nanoparticles purity percentages of wollastonite.

To evaluate the kind of acid during the formation of HF during the reaction with the CaF_2_, the effect of some acids (0.01 mol L^−1^) such as HNO_3_, HCl, HBr, HI, HIO_4_, H_2_SO_4_, H_3_PO_4_, and CH_3_COOH was evaluated in detail. For this purpose, the purity as well as the amounts of the synthesized wollastonite was evaluated using different analytical methods such as TGA. The results have been shown according to the histogram shown in Supplementary Fig. 1 (see Supplementary Fig. 1, Supplementary Information). As shown, the maximum purity percentage was observed during using HCl. Therefore, HCl was selected as the optimum acid species. In addition, the thermogram and the DSC curves related to the effect of HCl have been shown in Fig. 2. Based on the results, maximum quantity and purity percentages were observed when using the HCl solution. Therefore, this acid was selected as a suitable acid medium. This result also pointed to the formation of two strong and weak acids. It seemed that, the strong behaviour of HCl as well as the effect of Cl^-^ during the formation of the complex with Ca^2+^ was the main reason for this effect.

### Effect of molar ratios of CaF_2_:HCl

To estimate the probable product(s) during the addition of HCl to CaF_2_ with the same molar concentrations (0.01 mol L^−1^), changes in the of pH value were evaluated in detail. For this purpose, to optimize the pH value, different molar ratios of CaF_2_:HCl were evaluated. The results have been shown in Supplementary Fig. 2 (see Supplementary Fig. 2, Supplementary Information). As shown, increase in the mole ratio of the CaF_2_:HCl up to 1:4, resulted in significant change in the pH value of the solution. Whereas, no major change was observed during further increase in the mole ratio of CaF_2_:HCl versus that of 1:4. Consequently, a steady state condition was observed in the pH value from 1:4 mol ratio. That was in good agreement with that expected in the proposed mechanism for the wollastonite nanoparticles, based on the equation shown in the previous chapter (Eq. 2). Consequently, the volume and concentration of the HCl solution were adjusted based on the CaF_2_:HCl with a mole ratio of 1:4.

### Acid/base titrimetry

In this study, changes in the pH value of the solution during introduction of HCl were experimentally evaluated in detail using a calibrated pH meter (Metrohm). The results have been shown in Supplementary Fig. 3 (see Supplementary Fig. 3, Supplementary Information). As shown, the pH value was two promoted from 3.2 to 4.5 (± 0.1) after the addition of 10.0 mL HCl (0.01 mol L^−1^) that revealed the effective role of HCl for reaction with CaF_2_ and SiO_2_ during the formation of the wollastonite nanoparticles.

In addition, for further evaluation of the probable mechanism for the formation of HF and H_2_[CaCl_4_], a solution sample contained CaF_2_ and HCl mixture with 1:4 mol ratio was also titrated with standard NaOH solution (0.01 mol L^−1^) according to the titration curve shown in Supplementary Fig. 3 (see Supplementary Fig. 3, Supplementary Information).

As clearly shown (see Supplementary Fig. 3, Supplementary Information) observation of three independent endpoints was related to the formation of HF and H_2_[CaCl_4_] in the first step of the synthesis of the wollastonite nanoparticles. However, it should be noted that, due to the destructive effect of HF on the glass pH electrode during the pH-based potentiometric titration, a negative error was observed on the pH values. Also, to evaluate the acidity as well as the concentrations of the acids after finalizing the reaction, i.e. after 20 min form the beginning of the reaction, 1.0 mL of the aqueous solution was diluted 100 times and titrated with NaOH (0.1 mol L^−1^) as the titrant. The result has been shown in Supplementary Fig. 4 (see Supplementary Fig. 4, Supplementary Information). Based on the result (Supplementary Fig. 4), two independent endpoints were observed during the titration of the solution with NaOH standard solution. In addition, observation of a buffer condition at pH value around 6.0, clearly pointed to the presence of a third stable species in the solution. Also, the estimated molar concentrations of each HCl and HF with relative error percentage at ~ -6.67% pointed to the accuracy of the proposed mechanism.

### Effect of temperature

Due to the volatility of HCl and HF at high temperatures, room temperature was chosen as optimal.

### Effect of sonication

**Figure 3:**
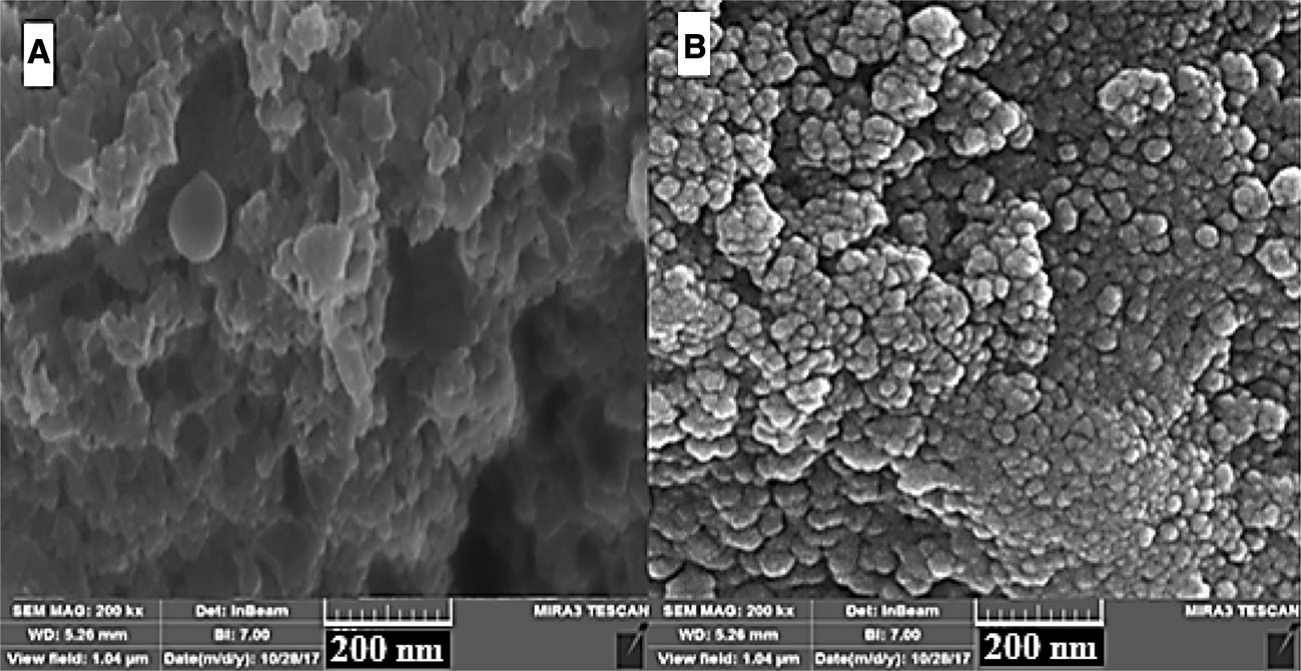
SEM images of the wollastonite nanoparticles (**A**) before and (**B**) after apply ultrasonic radiations at frequency around 3.2 MHz for 45.0 min.

Another important factor having a strong influence on the size and morphology of the wollastonite nanoparticles was the sonication. To evaluate this factor, the wollastonite nanoparticles were synthesized before and after irradiating the sonication radiations using a sonicator (UP200H-Ultrasonic Processor Hielscher, USA) with a frequency of 24 kHz for 45.0 min according to the high-resolution SEM shown in Fig. 3. About the SEM images, this magnification was selected to better observe the effective role(s) of the sonication on the macroscopic shaping of the tested supports. In another word, when using higher resolution images, the roughness as well as high active surface area of the synthesized wollastonite nanoparticles were so high that, the differences were not practically differentiated.

**Figure 4:**
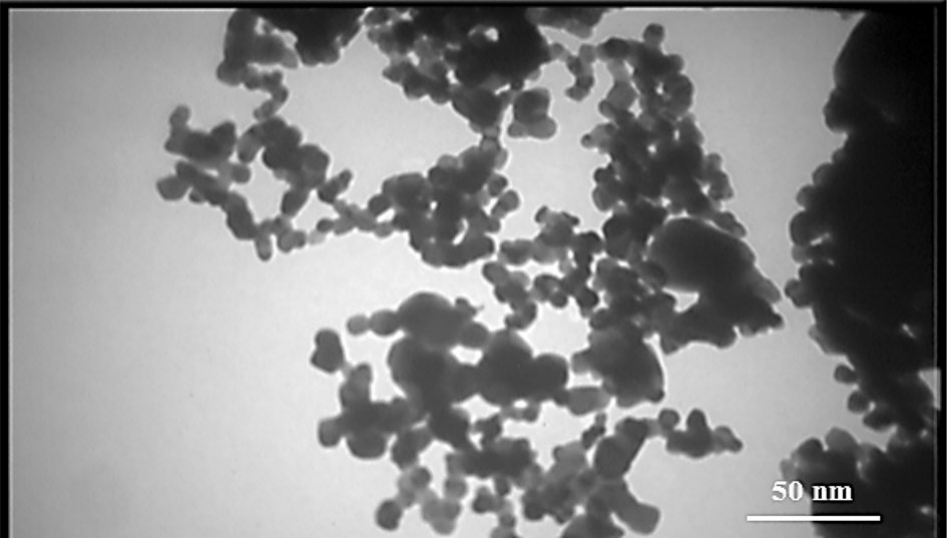
High resolution TEM image of the synthesized wollastonite nanoparticles.

As clearly shown (Fig. 3), the wollastonite sample before ultrasonic irradiation has been agglomerated and aggregated; whereas the average size of the wollastonite species during irradiating the ultrasonic radiations was estimated to be around 10 nm. As shown, ultrasonic irradiation led to synthesize wollastonite nanoparticles with nano-meter ranges with an average of around 10 nm. In addition, in spite of utilizing similar conditions for the both reported SEM images such as the identical too bar length (i.e. 200 nm), but, major resolution (more contrast) was observed for the wollastonite nanoparticles (Fig. 3B), relative to the agglomerated ones (Fig. 3A). It seemed that, the nano sizes of the wollastonite nanoparticles have played role as the active sites (defects) for higher resolute electron-scanning process during the SEM imaging. Consequently, this method can be considered as a simple and novel technique for large-scale synthesis of the wollastonite nanoparticles. In addition, an acceptable correlation was observed between the SEM image (Fig. 3) and the high-resolution transition electron microscopic (HR-TEM, Zeiss EM10, 20 kV) image, Fig. 4).

According to the HR-TEM showing in Fig. 4, the histogram related to the size distribution of the synthesized wollastonite nanoparticles has been shown in Supplementary Fig. 5 (see Supplementary Fig. 5, Supplementary Information). Narrow size distribution was observed between 2 and 25 nm for the synthesized wollastonite nanoparticles. As conclusion, both macroscopic (SEM images, Fig. 3) and HR-TEM (Fig. 4) images reveal the normal size distribution of the synthesized wollastonite nanoparticles.

### FT-IR characterization of wollastonite nanoparticles

**Figure 5:**
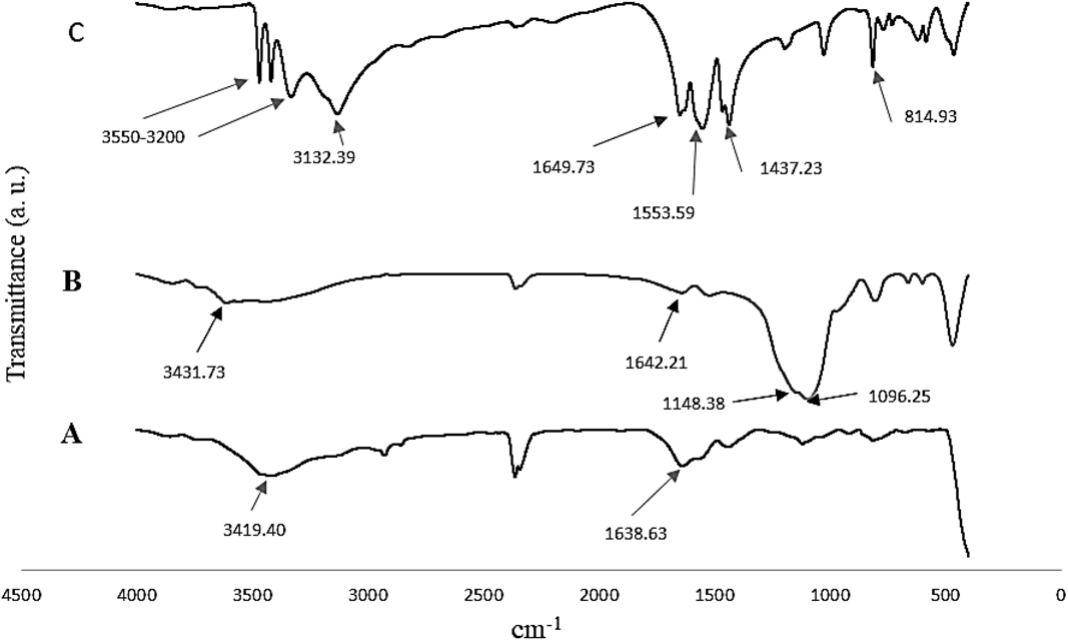
FT-IR spectra of the CaF_2_ (**A**), SiO_2_ (**B**) and the synthesis wollastonite nanoparticles (**C**).

The FT-IR spectra of the CaF_2_, SiO_2_ and the synthesized wollastonite nanoparticles have been shown in Fig. 5.

Figure 5A is related to the FT-IR spectrum of CaF_2_. As shown no absorption was observed that reveals the lack of any functional groups in the CaF_2_ matrix at the mid- IR region. Whereas, the peak positioned at around 1130 cm^−1^ is attributed to the Si–O–Si functional group of the SiO_2_ (Fig. 5B). In addition, based on the results shown in Fig. 5C, the synthesized wollastonite nanoparticles possess different functional groups such as O–H at frequencies ranged between 3100 and 3550 cm^−1^. In addition, the peaks, positioned at 1437.23, 1553.59 and 1649.73 cm^−1^ were attributed to the Si=O functional group. The absorption peak related to the Ca–O functional group was observed at frequencies around 814.93 cm^−1^^9^. These functional groups were considered as the ones belong to the wollastonite compound^10^^,17^. High purity of the wollastonite nanoparticles was also confirmed based on the lack of observing any unrelated and undefined functional groups according to the FT-IR spectra (Fig. 5).

### XRD characterization of wollastonite nanoparticles

**Figure 6:**
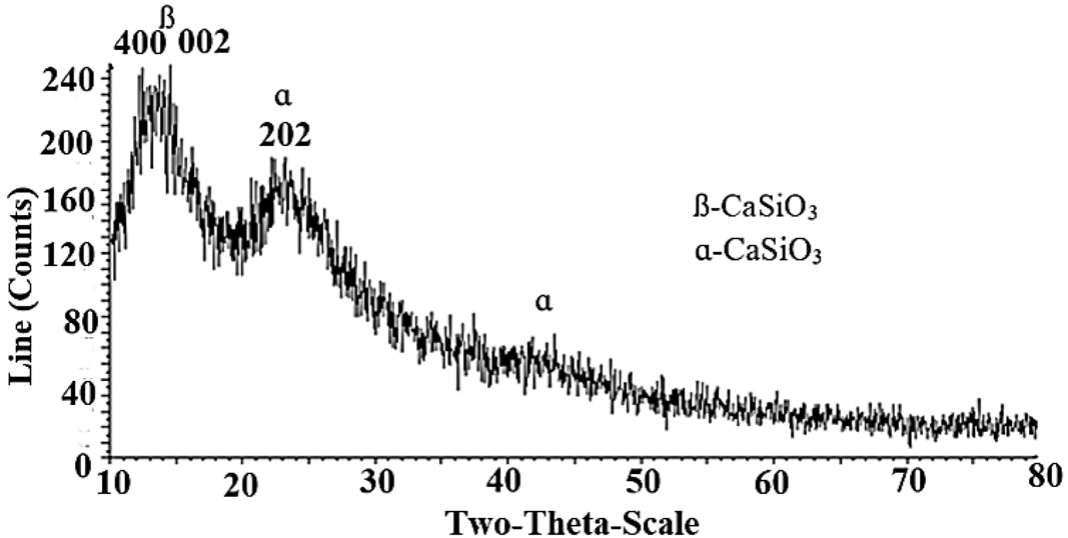
Patterned XRD of the synthesized wollastonite nanoparticles.

The XRD pattern of the wollastonite sample is shown in Fig. 6. This pattern shows two different morphologies including alpha and beta for the synthesized wollastonite nanoparticles according to the recommended procedure.

Based on the XRD pattern (Fig. 6), the main peaks of 2θ positioned at 14 and 23° is belong to the (400), (002), and (202) morphologies of each α- and *ß*-CaSiO_3_^10^. In addition, the presence of no sign about the possibility of the formation of other compounds such as Ca_2_SiO_4_ (at 2θ =  ~ 30°)^10^, points to high purity of the synthesized wollastonite nanoparticles. Based on the XRD database as well as the literature review studies, the lack of observation of any extra morphologies in the XRD pattern such as those related to the carbonation process, calcite, unreacted, etc.^17^, reveals the potential of this method to synthesize wollastonite nanoparticles with high enough purity percentage. Therefore, partial difference between Fig. 6 and the previously reported XRD patterns, further exhibits the priority of this method from the purity aspect as well as accuracy of the recommended mechanism and optimization process. The morphology of the highly pure wollastonite nanoparticles is therefore confirmed by the recommended procedure.

### Mixed matrix anti-corrosive experimental design

Design of the wollastonite-based MM as anti-corrosive support is based on mechanical stability and some electrochemical analyses such as linear polarization. About this MM, graphite is considered as a necessary binder for promotion of the mechanical stability of the synthesized MM. Based on the direct observations, no significant connectivity is observed in the absence of any graphite component. Besides this, presence of graphite as conductive material promotes the electrical conductivity of the MM. In the absence of graphite powder, the electrical resistivity of the composite is so high that, it is practically impossible to electrochemically follow the corrosion current versus time. Consequently, graphite powder is considered as one of the main share of the MM sample.

In addition, wollastonite nanoparticle plays role as another important part (filler) during designing the MM. This claim is based on the following the linear polarization of the MM in the presence and absence of wollastonite nanoparticles under similar conditions (Supplementary Fig. 6, Supplementary Information). Based on the results, presence of wollastonite nanoparticle in the MM during immobilizing on Fe rod as the working electrode, resulted in significant reduction and enhancement in the *i*_*corr*._ and *E*_*appl*._, respectively. However, due to the no electrical conductivity of the epoxy/melamine formaldehyde composite, it was impossible to evaluate its anti-corrosive properties; whereas, relative to the epoxy/graphite/melamine formaldehyde composite, the novelty of the wollastonite nanoparticles was further confirmed when evaluating the anti-corrosive properties of the Fe electrode-modified epoxy/graphite/wollastonite/melamine formaldehyde /melamine formaldehyde MM (Supplementary Fig. 6). Consequently, wollastonite nanoparticle is selected as an effective filler during observing the anti-corrosive property in the synthesized MM. However, wollastonite nanoparticle plays significant role on the promoting the corrosion resistance of synthesized MM material. Therefore, besides the graphite powder, the weight percentage of the wollastonite nanoparticles controls the mechanisms stability of the synthesized MM. Conversely, insulation property of the further amounts of wollastonite nanoparticle in the composite matrix has significant effect on the promotion of the corrosion resistance. This result points to the importance of the optimization of the weight percentage of the synthesized composite.

However, the homogeneity of the dispersion (mixing) of the wollastonite nanoparticle as nano-filler during fabrication of the MM evaluated via following the standard deviation of the i_corr._ and E_appl_ along replicate testing of different MM-based samples at different time periods or during sampling from the different parts of the synthesized MM sample. In this study, acceptable standard deviation (< 4.0%, n = 3) reveals the homogeneity of the different phase of the MM by the recommend procedure.

### Optimization of coating percentage

To estimate the weight percentage (wt%) of each component of the MM sample, some different proportions of dissimilar weight-matrix components were evaluated in detail. Based on the measured electrical current as well as an increase in the electrical potential, the weight percentages of coating were optimized by the one-at-time method. Optimized values related to the wt. % of each component, used in the fabrication of the MM as anti-corrosive coating at different environments were reported in Supplementary Table 2 (see Supplementary Table 2, Supplementary Information). It should be noted that, this optimization was performed via following the linear polarization of the synthesized MM in the acidic and salt environments (Supplementary Table 3, Supplementary Information).

As shown, after a conductive solid support is coated and modified with the fabricated MM, it should be mixed with the epoxy polymer to obtain a homogeneous solid mixture. Then, related hardener is added (v/w %) to the previously provided composite with a ratio of 30.0–100.0% (w/w). To study the electrochemical methodologies on the synthesized MM, it is then modified on the surface of electrical supports such as a Fe or steel rod. After applying the backing process, it is used as a modified working electrode according to the recommended procedure. It is also necessary to cover a layer of coating on the electrode surface during cooking the surface at 100–200 °C in a vacuum oven for 3.0 h, based on the reported procedure.

The SEM images of the synthesized wollastonite nanoparticle from different locations have been shown in Supplementary Fig. 7 (see Supplementary Fig. 7, Supplementary Information). This magnification was selected to better evaluate the macroscopic (global) imaging the surface versus corrosion phenomenon. As clearly shown, high active surface area (Supplementary Fig. 7, Parts: a–c), lots of surface defects (active sites, Supplementary Fig. 7, Parts: a–f), multipole void volumes (Supplementary Fig. 7, Part: e) and enough flexibility and selective permeability (due to the formation of cracks (gaps, Supplementary Fig. 7, Parts: b, c and f) were illustrated for the synthesised MM. However, little smooth parts were observed on the synthesized MM (For example, Supplementary Fig. 7, Part: d), acceptable features was detected for this compound. These characteristics pointed to the suitability of the MM coating for providing ideal anti-corrosive properties such as enough active surface area, blocking/limiting the physical contacts between the corroding species and the steel, resistance to the pitting, possibility to the surface storage for long time, large anti-corrosive efficacy, slow corrosion rate, etc.^8^. Consequently, no significant corrosion is detected after its coating on the surface of the electroactive supports. These results shows the MM to have anti-corrosive property.

### Linear polarization and corrosion rate

The electrochemical parameters such corrosion current density (*i*_*corr*_), corrosion potential (*E*_*corr*_) and corrosion rate (*C.R.*) values were calculated vs. potentiodynamic polarization measurements of iron at different environments such as 1.0 mol L^−1^ HCl and 3.0 wt% (w/v) NaCl solutions. The results were shown in Supplementary Table 3 (see Supplementary Table 3, Supplementary Information). In this study, the corrosion rate (*C.R*) in MPY (mils penetration per year) is calculated using (Eq. 5):^8^

5 $$ C.R. = K\frac{ai}{{nD}}, $$

where “*a*” is the atomic weight of the metal, “*i*” is the current density in A cm^-2^, "*n*" is the number of electrons lost, "*D*" is the density in g.cm^-3^ and *"K"* is a corrosion constant depending on the unit of corrosion rate^8^.

By performing a weight-loss test, it was found that, the coating had a very high corrosion resistance. This effect could be seen based on the results shown in Supplementary Table 4 (see Supplementary Table 4, Supplementary Information). By comparing the *C.R. %* in Supplementary Table 4 (see Supplementary Table 4, Supplementary Information), the *C.R. %* were found as 74.86% for the steel in the absence of any MM coating. While, the corrosion rate *%* was majorly decreased from 74.86 to 0.34% during modification of the steel with the MM. These results could be further confirmed via observation through the optical microscope images (Supplementary Figs. 8 and 9, Supplementary Information). All this evidences, therefore point to the efficient anti-corrosive behaviour of the introduced MM.

### Effect of environments on the anti-corrosive behaviour of the fabricated mixed matrix

To study the effect of different environments on the performance of the introduced MM at the optimum conditions, the effect of different types of MM-modified Fe or steel-based electrode was evaluated in detail. The linear polarization curves have been shown in Supplementary Fig. 10 (see Supplementary Fig. 10, Supplementary Information). As clearly shown, an acceptable shift was observed in the electrical potential and current that revealed good performance of the introduced MM for playing the role as an anti-corrosive coating at different hard environments especially H^+^ and Cl^−^ with high molar concentrations.

Fortunately, the fabricated MM was also applicable inside the non-acidic environments such as NaCl and KCl with significant potentials shift from − 625.0 to 650.0 and from − 508.0 to − 540.0 mV (vs. Ag/AgCl) for each NaCl and KCl solution, respectively; at similar conditions according to the linear polarization curves.

During testing the morphology of the Fe surface before and after modifying with the coating process, it was concluded that, the coated metal had a high corrosion resistance. As the SEM images show (Supplementary Fig. 11, Supplementary Information), the metal surface after applying with the electrical potential by the CV mode in the NaCl solution with 0.5 mol L^−1^ concentration showed severe pitting corrosion. Compared to the uncoated Fe electrode, under the same conditions, the coating had been able to protect the Fe metal surface effectively from any types the corrosion phenomenon (Supplementary Fig. 11, Parts d, e, f, and g, and Supplementary Information).

### Electrochemical impedance spectroscopic measurements (EIS)

Electrochemical impedance spectroscopy (*EIS*) was also considered as another important technique for evaluation of further evaluation of the double-layer resistance (*R*_*dl*_) of the electrode system in the presence or absence of the anti-corrosive MM coating at different environments like H^+^ or Cl^−^ provided using various reagents (Supplementary Fig. 12, Supplementary Information). It should be noted that, unfortunately, due to the presence of sophisticated multi-processes in the corrosive field as well as the strong relationship between this phenomenon and the environments, it was imposable to completely fit a reliable equivalent circuit for the Nyquist plots using the existing equivalent circuit models in the database on the “*Metrohm*” instrumentation system.

However, besides the change in the characteristics of the responsible elements such as resistance, capacitor, and self (Supplementary Fig. 12, insets, Supplementary Information) before and after modification with the MM, major enhancements were observed for the *R*_*dl*_. This was according to the limit of the curvature of the Nyquist plots that again revealed the acceptable anti-corrosive behaviour of the MM. In addition, the electrical conductivity of the MM was again evidenced during focusing on the solution resistance (*R*_*s*_) according to the beginning parts of the Nyquist plots^11^.

### Weight-loss measurements

For finalizing the evidence about the anti-corrosive behaviour of any coating, weight-loss measurements over a long time such as a 42 day time period, have also been recommended. To do this test, two pieces of stainless steel plates (Tip: St-52, characterized in Supplementary Table 1, see Supplementary Information) were selected with dimensions as large as 10 × 20 × 2.0 mm. For this purpose, one plate was considered as the control and the other one was modified with the synthesized MM. After the introduction of a hard condition such as 100.0 mL HCl solution with 1.0 mol L^−1^ concentration during the 42 days inside a close bottle at room temperature (according to the procedure reported in Ref.^12^) under similar condition, the results have been reported in Supplementary Table 4 (see Supplementary Table 4, Supplementary Information).

## Conclusions

In this study, a novel method was presented for the synthesis of wollastonite nanostructures, which had advantages such as simplicity, low cost, as well as no need to high temperatures and pressures. The use of these wollastonite nanostructures as a nano-filler in the MM, led to the emergence of a new category of polymer coatings that behaved as acceptable anti-corrosive coating with highly enough electrical resistance against any corrosion(s) with high mechanical and thermal stability. In addition, this coating could widely be used as a dye paint for metal surfaces and as well as for the cement replacement. This coating had also high enough adhesion property. Consequently, the MM support was considered as an environmentally green coating, due to its acceptable environmental compatibility. This coating also reduced the extents surface oxidation and reduction reactions along with the reduction of the corrosion rate significantly. As explained in detail in the weight-loss tests, when the MM coating was applied on any type of alloy surface, it caused a significant reduction in the corrosion rate as from 74.86 (for the bared stainless steel) to 0.34% (for the MM-modified stainless steel) after 42 days. The final talk is that, the acceptable and applicable effective role of the introduced MM as the anti-corrosive coating is concluded.

## Supplementary Information

Supplementary Information.
