# Supplementary material for: Novel method for scalable synthesis of wollastonite nanoparticle as nano-filler in composites for promotion of anti-corrosive property
Source: Sci Rep. 2021 Jan 28;11:2579. doi: 10.1038/s41598-021-81875-4 (PMC7843617; doi:10.1038/s41598-021-81875-4)
Supplement: Supplementary file 1 — Supplementary Information. [file 41598_2021_81875_MOESM1_ESM.docx]

Supporting Information

Related to

**Novel Method for Scalable Synthesis of Wollastonite Nanoparticle as nano-filler in Composites for Promotion of Anti-Corrosive Property**

Rouholah Dordane, Mohammad Mahdi Doroodmand *

Content:

*Figures*

**Figure 1.SP |** Histogram showing the effect of different acids (0.01 mol L^-1^) on the purity percentage of the wollastonite nanoparticles using the thermo-gravimetric analysis (n=3).

**Figure 2.SP |** Histogram showing the correlation between pH and the different mole ratios of HCl: CaF_2_ (n=3).

**Figure 3.SP |** Titration curve of HF and H_2_ [CaCl_4_] with NaOH (0.01 mol L^-1^).

**Figure 4.SP |** Titration curve of the HF and HCl as the product of the wollastonite nanoparticles using NaOH (0.1 mol L^-1^) as the titrant.

**Figure 5.SP |** Histogram showing the diameter range of the synthesized wollastonite nanoparticles. The sizes are the average of three independent tests.

**Figure 6.SP |** Linear polarization of A) bare Fe electrode, B) Fe electrode modified with epoxy/graphite/melamine formaldehyde and C) Fe electrode modified with epoxy/graphite/ Wollastonite/melamine formaldehyde in 3.0 % NaCl environment.

**Figure 7.SP |** SEM images fabricated MM from different locations.

**Figure 8.SP |** Optical microscopic images of a bare and modified stainless steel a) fresh and b) MM-modified stainless steel plats as well as c) and d) those, respectively contacted with a solution of HCl with 1.0 mol L^-1^ concentration for 42 days.

**Figure 9.SP |** Optical microscopic images of a bare and modified stainless steel a) fresh and b) MM-modified stainless steel plats as well as c) and d) those, respectively contacted with a solution of HCl with 1.0 mol L^-1^ concentration for 42 days with different magnifications and from different sides of view.

**Figure 10.SP |** Linear polarization of 1) Fe electrode and 2) MM-modified Fe electrode in A) HCl environment with 1.0 mol L^-1^ concentration, B) HNO_3_ environment with 1.0 mol L^-1^ concentration, C) citric acid environment with 5.0 % (w/w) concentration, and D) NaCl 3.0 % (w/w).

**Figure 11.SP |** SEM images a) fresh Fe electrode, b) fresh MM-modified Fe electrode, as well as c) and d) those, respectively applied with the CV mode using NaCl with 0.5 mol L^-1^ concentration electrolyte. Notations e, f, g, and h point to other parts of the coating and the metal electrode surface.

**Figure 12.SP |** Nyquist plots including A) Fe and B) MM-modified Fe electrodes using the three-electrode system inside 1, 2) citric acid 5.0 % (w/v) environment 3, 4) HNO_3_ environment with 1.0 mol L^-1^ concentration, 5) and 6) KCl environment with 3.0 % (w/v) concentration during scanning of the frequency from 0.1 MHz to 0.1 Hz. Inset) Bode plots. Insets: Bode plots.

***Tables***

**Table 1.SP |** Chemical composition of stainless steel and iron using Quantometry (model: Foundry Master Smart).

**Table 2.SP |** Optimized values related to the wt.% of each component, used in the fabrication of the MM as anti-corrosive coating at different environment.

**Table 3.SP |** Experimental electrochemical parameters for corrosion of iron different environments by polarization method using the µ-AUTO LAB instrument (µ3AUT70980).

**Table 4.SP |** Results of weight-loss measurement.


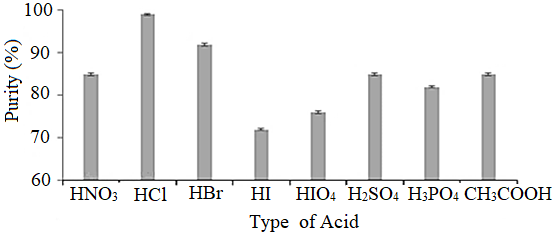


**Figure 1.SP |** Histogram showing the effect of different acids (0.01 mol L^-1^) on the purity percentage of the wollastonite nanoparticles using the thermo-gravimetric analysis, error bar: ± standard deviation (n=3).

**
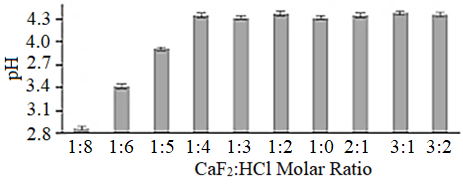
**

**Figure 2.SP |** Histogram showing the correlation between pH and the different mole ratios of HCl: CaF_2_, error bar: ± standard deviation (n=3).


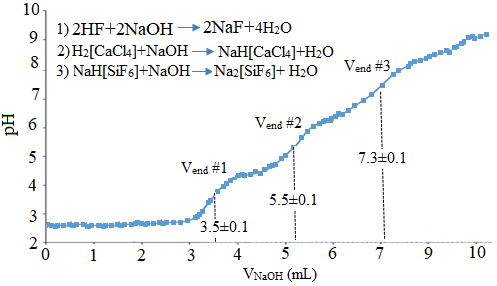


**Figure 3.SP |** Titration curve of HF and H_2_ [CaCl_4_] with NaOH (0.01 mol L^-1^).

**
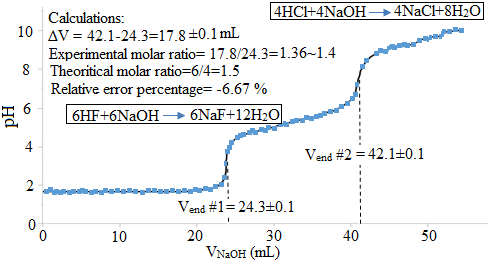
**

**Figure 4.SP |** Titration curve of the HF and HCl as the product of the wollastonite nanoparticles using NaOH (0.1 mol L^-1^) as the titrant.


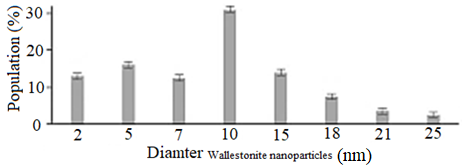


**Figure 5.SP |** Histogram showing the diameter range of the synthesized wollastonite nanoparticles. The sizes are the average of three independent tests, error bar: ± standard deviation.


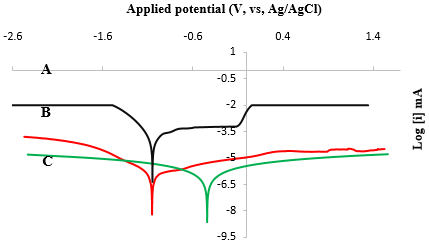


**Figure 6.SP |** Linear polarization of A) bare Fe electrode, B) Fe electrode modified with epoxy/graphite/melamine formaldehyde and C) Fe electrode modified with epoxy/graphite/ Wollastonite/melamine formaldehyde in 3.0 % NaCl environment.


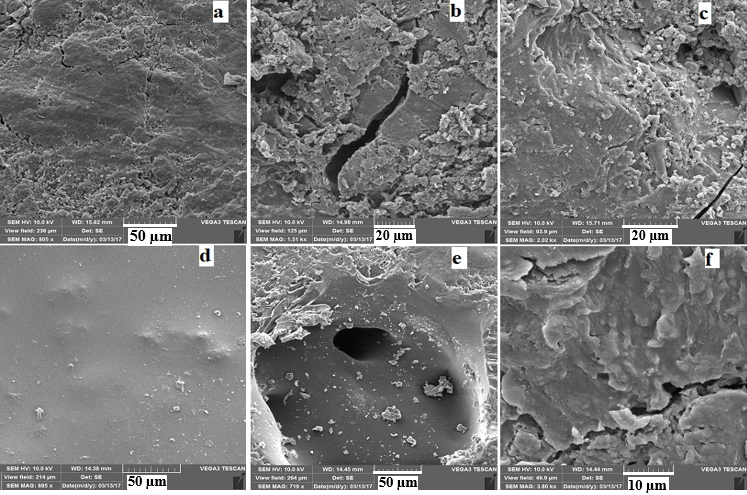


**Figure 7.SP |** SEM images fabricated MM from different locations. The SEM image of the synthesized wollastonite from different sides has been shown.


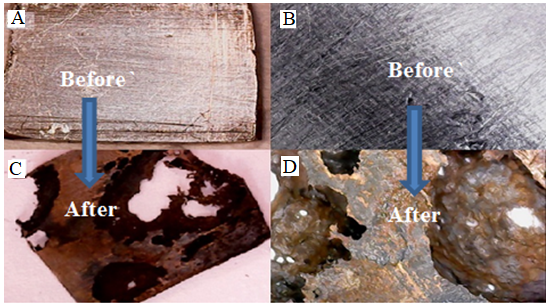


**Figure 8.SP |** Optical microscopic images of a bare stainless steel a) and b) fresh stainless steel plats as well as c) and d) those, respectively contacted with a solution of HCl with 1.0 mol L^-1^ concentration for 42 days with different magnifications and from different sides of view.


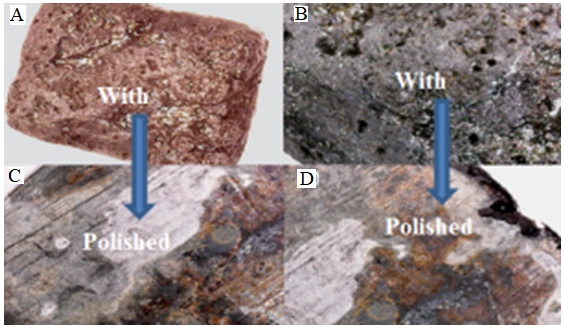


**F**

**Figure 9.SP |** Optical microscopic images of a modified stainless steel a) and b) fresh MM-modified stainless steel plats as well as c) and d) those, respectively contacted with a solution of HCl with 1.0 mol L^-1^ concentration for 42 days with different magnifications and from different sides of view.


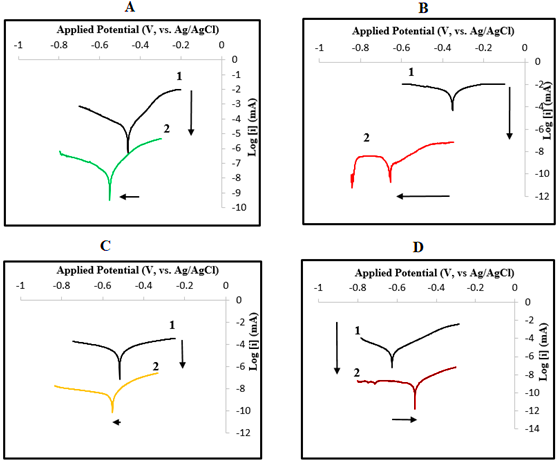


**Figure 10.SP |** Linear polarization of 1) Fe electrode and 2) MM-modified Fe electrode in A) HCl environment with 1.0 mol L^-1^ concentration, B) HNO_3_ environment with 1.0 mol L^-1^ concentration, C) citric acid environment with 5.0 % (w/w) concentration, and D) NaCl 3.0 % (w/w).

**
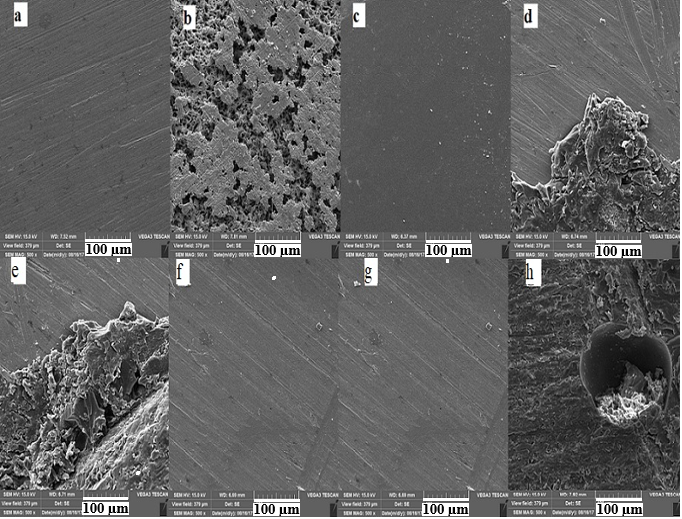
**

**Figure 11.SP |** SEM images a) fresh Fe electrode, b) fresh MM-modified Fe electrode, as well as c) and d) those, respectively applied with the CV mode using NaCl with 0.5 mol L^-1^ concentration electrolyte. Notations e, f, g and h point to other parts of the coating and metal electrode surface.


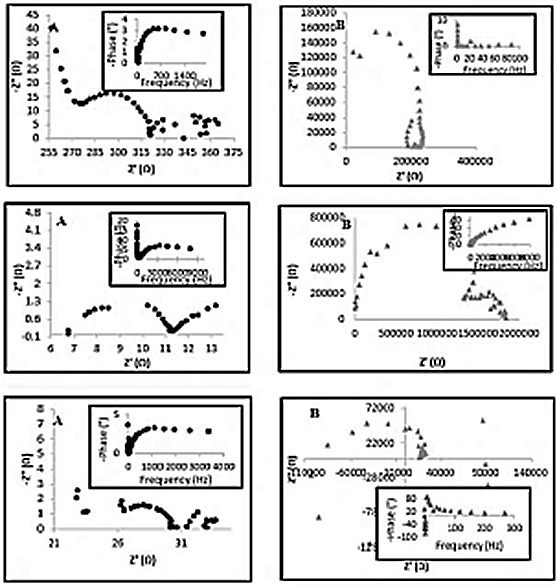


**Figure 12.SP |** Nyquist plots including A) Fe and B) MM-modified Fe electrodes using the three-electrode system inside 1, 2) citric acid 5.0 % (w/v) environment 3, 4) HNO_3_ environment with 1.0 mol L^-1^ concentration, 5) and 6) KCl environment with 3.0 % (w/v) concentration during scanning of the frequency from 0.1 MHz to 0.1 Hz. Inset) Bode plots. Insets: Bode plots.

| **Table 1.SP \|** Chemical composition of stainless steel and iron using Quantometry (model: Foundry Master Smart). | | | | | | | | | | | |
| --- | --- | --- | --- | --- | --- | --- | --- | --- | --- | --- | --- |
| Iron analysis | | | | | | | | | | | |
| *Element | Fe | O | Mn | Si | C | Cr | Ni | Al | Cu | S | Another |
| Wt% | 95.60 | 4.12 | 0.01 | 0.005 | 0.01 | 0.003 | 0.01 | 0.001 | 0.005 | 0.005 | <0.005 |
| Stainless steel | | | | | | | | | | | |
| *Element | Fe | Mn | Si | C | Cr | Ni | Al | Cu | S | P | Another |
| Wt% | 98.70 | 0.91 | 0.18 | 0.021 | 0.013 | 0.028 | 0.021 | 0.024 | 0.008 | 0.013 | <0.005 |

| **Table 2.SP \|** Optimized values related to the wt.% of each component, used in the fabrication of the MM as anti-corrosive coating at different environment.^1, 2^ | | | | |
| --- | --- | --- | --- | --- |
| **Environmental condition** | **Epoxy resin (w/w )** | **Acidic Melamine Formaldehyde (w/w)** | **Wollastonite (w/w )** | **Graphite (w/w )** |
| Cl^-^ environment | 65.0 | 15.0 | 10.0 | 10.0 |
| H^+^ environment | 65.0 | 10.0 | 15.0 | 10.0 |
| ^1^Data are the average of 3 independent analyses. ^2^ ±Standard deviations all the masses: 0.010 ± 0.001 mg. | | | | |

| **Table 3.SP \|** Experimental electrochemical parameters for corrosion of iron different environments by polarization method using the µ-AUTO LAB instrument (model: µ3AUT70980). | | | |
| --- | --- | --- | --- |
| ***Environment:*** 1.0 mol L^-1^ HCl solution | | | |
| **Condition** | ***i_corr_* (A)** | ***E_corr_* (mV)** | ***C. R* (mil/year)** |
| Without coating | (63.9270 ± 0.0001) × 10^-6^ | -461.02 ± 0.01 | 0.7428 |
| With coating | (24.0050 ± 0.0001) × 10^-9^ | -550.33 ± 0.01 | 0.0003 |
| ***Environment:*** NaCl 3% (v/w) | | | |
| **Condition** | ***i_corr_* (A)** | ***E_corr_* (mV)** | ***C.R* (mil/year)** |
| Without coating | (11.3840 ± 0.0001) × 10^-6^ | -624.80 ± 0.01 | 0.1323 |
| With coating | (1.1734 ± 0.0001) × 10^-9^ | -508.68 ± 0.01 | 1.3635 × 10^-5^ |
| The data are the average of 3 independent analyses with an error bar of ± standard deviation. C. R: is corrosion rate | | | |

| **Table 4.SP \|** Results of weight-loss measurement. | | | | |
| --- | --- | --- | --- | --- |
| **Type** | ***W_1_***  **(mg)** | ***W_2_***  **(mg)** | **Weight changes after 42 days (mg)** | ***C.R***  **(%)** |
| **Fresh stainless steel** | 2504.90 ± 0.01 | 629.80 ± 0.01 | 1875.10 ± 0.01 | 74.86 ± 0.01 |
| **MM-modified stainless steel** | 2283.48 ± 0.01 | 2275.8 ± 0.01 | 7.68 ± 0.01 | 0.34 ± 0.01 |
| Data are the average of three independent analyses. Error bars were reported based on the standard deviation. | | | | |
